# Passive Immunization with Hypochlorite-oxLDL Specific Antibodies Reduces Plaque Volume in LDL Receptor-Deficient Mice

**DOI:** 10.1371/journal.pone.0068039

**Published:** 2013-07-16

**Authors:** Marcella van Leeuwen, Michael J. Kemna, Menno P. J. de Winther, Louis Boon, Adriaan M. Duijvestijn, Darius Henatsch, Nico A. Bos, Marion J. J. Gijbels, Jan Willem Cohen Tervaert

**Affiliations:** 1 Internal Medicine, Clinical and Experimental Immunology, Cardiovascular Research Institute Maastricht, Maastricht University Medical Center, Maastricht, The Netherlands; 2 Molecular Genetics, Cardiovascular Research Institute Maastricht, Maastricht University Medical Center, Maastricht, The Netherlands; 3 Department of Medical Biochemistry, Academic Medical Center (AMC), University of Amsterdam, Amsterdam, The Netherlands; 4 Bioceros B.V., Utrecht, The Netherlands; 5 Department of Rheumatology and Clinical Immunology, University of Groningen, University Medical Center Groningen, The Netherlands; 6 Pathology, Cardiovascular Research Institute Maastricht, Maastricht University Medical Center, Maastricht, The Netherlands; 7 Immunology Laboratory, Maastricht University Medical Center, Maastricht, The Netherlands; University of Amsterdam Academic Medical Center, The Netherlands

## Abstract

**Aims:**

New strategies to overcome complications of cardiovascular diseases are needed. Since it has been demonstrated that atherosclerosis is an inflammatory disease, modulation of the immune system may be a promising approach. Previously, it was suggested that antibodies may confer protective effects on the development of atherosclerosis. In this study, we hypothesised that passive immunization with anti-oxLDL IgM antibodies specific for hypochlorite (HOCl) may be athero-protective in mice.

**Methods and Results:**

Monoclonal mouse IgM antibodies were produced and the antibody with specificity for hypochlorite-oxLDL (HOCl-oxLDL) (Moab A7S8) was selected. VH sequence determination revealed that Moab A7S8 is a natural IgM antibody. Atherosclerosis in LDLr^−/−^ mice was induced by a perivascular collar placement around the right carotid artery in combination with feeding a high-fat diet. Subsequently, the mice were treated every six days with 500 µg Moab A7S8, non-relevant IgM or with PBS and the carotid arteries and aortic roots were studied for atherosclerosis. Passive immunization with this Moab A7S8 resulted in a significant reduced plaque volume formation in LDLr^−/−^ mice when compared with PBS treatment (P = 0.002 and P = 0.035). Cholesterol levels decreased by 20% when mice were treated with Moab A7S8 compared to PBS. Furthermore, anti-oxLDL specific IgM and IgG antibody production increased significantly in the Moab A7S8 treated mice in comparison with PBS treated mice.

**Conclusion:**

Our data show that passive immunization with a natural IgM antibody, directed to HOCl-oxLDL, can reduce atherosclerotic plaque development. We postulate that specific antibody therapy may be developed for use in human cardiovascular diseases.

## Introduction

Atherosclerosis is the most important underlying cause of cardiovascular diseases and is a major contributor of morbidity and mortality in the western society. In large randomized clinical trials complications such as myocardial infarction and stroke, are reduced by less than 50% with current therapy. Therefore, development of novel therapeutic strategies is highly needed to complement or replace current treatments [1]. Both the cellular and humoral immune responses has been increasingly recognized as essential in atherogenesis [2].

Immune-modulation therapy via a passive immunization strategy aims to exploit the athero-protective aspects of the immune system to modulate the development of atherosclerosis [3], [4]. It was demonstrated in a vein graft atherosclerosis model that passive immunization with T15 natural IgM antibodies could reduce plaque development by 25% [5]. This suggests a potential role for IgM antibodies in passive immunization strategies. T15 IgM antibodies are considered to be part of the innate immune response which are of natural origin. These antibodies are secreted by distinct sets of innate-like B cells, B−1 cells and marginal zone B cells, which arise early in development and become the source of “natural immune memory”. Due to their interactions with a variety of self-determinants, natural antibodies have previously been postulated to be important for the maintenance of host homeostasis [6], [7]. Oxidation derived epitopes on apoptotic cells and on LDL (oxLDL) are recognized by the phosphorylcholine (PC) – specific encoded B−1 cell natural T15 antibody [6]. *In vitro*, IgM autoantibodies to oxLDL block the binding and degradation of oxLDL by macrophages [8]. Increased levels of natural IgM T15 antibodies with specificity for the phosphorylcholine epitope provide a protective effect in mouse atherosclerosis as shown by a reduction of atherosclerotic plaque formation [9], [10]. Moreover, high levels of immunoglobulin M type of autoantibodies against phosphorylcholine were found to be protective against human atherosclerosis [11]–[13], the latter was also found to be a prognostic factor in acute coronary syndromes [14].

OxLDL plays a pivotal role throughout development of atherosclerosis. Modification of LDL into its oxidized form is caused by several different mechanisms. One clinically relevant pathway is via myeloperoxidase (MPO) and its oxidant product hypochlorite (HOCl) [15], [16]. Active MPO can be demonstrated in extracts from human atherosclerotic arteries [17], circulating MPO levels independently predict the risk to develop events of cardiovascular diseases [18], [19] and −463 MPO polymorphism predicts the risk for cardiovascular events [20]. In mice and humans, increased titers of autoantibodies against HOCl-oxLDL have been reported during atherogenesis [4], [21]–[23]. Recently, we demonstrated presence of neutrophils with co-localized MPO in mouse atherosclerotic plaques [24]. Furthermore, increased levels of circulating MPO were observed in atherosclerosis prone mice upon high-fat feeding [24].

We hypothesised that atherosclerotic plaque development can be reduced with passive immunization of IgM antibodies specific for HOCl-oxLDL. To test this hypothesis, we selected a monoclonal antibody that bound to hypochlorite-oxLDL and performed passive immunizations in our LDLr^−/−^ mouse model for atherosclerosis.

## Materials and Methods

### Ethics Statement

The investigation conforms to the Guide for the Care and Use of Laboratory Animals published by the directive of the European Parliament (Directive 2011/63/EU). Approval was granted by the ethics review board of Maastricht University (reference number: 2008–111) and carried out in compliance with the Dutch government.

### Immunization and Culture of Hybridoma Cell Line

Balb/C mice were immunized with hypochlorite-modified oxLDL [21]. Mice received an intraperitoneal dose of 50 µg of oxLDL in 100 µl sterile-filtered PBS and an equal volume of Complete Freund`s Adjuvant on day 0, followed on day 21 and 35 by booster injections of 50 µg oxLDL in Incomplete Freund`s Adjuvant. Five days before sacrifice, 50 µg of oxLDL was administered intravenously. At day 48, mice were sacrificed and spleens were harvested. A single cell suspension was prepared and fused with the HAT-sensitive cell line SP_2_O. Anti-oxLDL IgM producing clones were selected by enzyme-linked immunosorbent assay (ELISA) as described before [22].

### Generation and Purification of IgM Antibodies

Antibody-containing medium was generated by culturing the hydridoma cell line in IMDM containing 1% FCS. To IgM-containing medium, 0.75 M of amoniumsulfate was added and the medium was loaded on a thiophilic agarose resin. The column was eluded using a 50 mM Tris-HCl buffer at pH 8.9. Protein concentration was determined as absorption at A280. Purified IgM antibodies were dialysed against PBS and subsequently filtered by a 0.2 µM filter and stored at −80°C.

### Characterization of Anti-oxLDL IgM Antibody

#### Western blotting

Purified monoclonal IgM antibody recognizing HOCl-modified LDL was obtained as described above. The antibody preparation was dissolved in non-reducing sample buffer and analyzed by SDS- Poly-Acrylamide-Gel Electrophoresis (SDS-PAGE), using a modification of the method described by Laemmli [25]. Ten µg protein were run on a 2–10% gradient gel for four hours at maximal 400 volt, and proteins were transferred to nitrocellulose membrane (Schleicher Schuell, Keene, NH) during one hour at 70 V. Following blocking with NaCl/Tris buffer containing 1% bovine serum albumin (BSA; Merck, Darmstadt, Germany), blots were incubated with anti-IgM antibody coupled to alkaline phosphatise; staining was visualized using an AP vector kit (Vectot Laboratories, Burlingame, CA) according to the manufacturer’s instructions.

#### Mouse monoclonal antibody isotype determination

The one-step mouse monoclonal antibody determination (Mouse mAb isotyping kit, HBT, Uden, The Netherlands) procedure involves the capture of the mouse immunoglobulins by subclass specific rat anti-mouse monoclonal antibodies which are immobilized on the test strip.

#### Reactivity of monoclonal IgM with LDL and oxLDL

LDL was isolated according to Smook et al. [26] Shortly, native LDL and hypochlorite oxidized LDL (HOCl) was coated in a concentration of 100 µg/ml in PBS, in microtitre plates (Nunc MaxiSorp™, Nalgene Nunc, Rochester, NY) overnight at 4°C. Wells were then washed five times with a buffer containing 0.01 M Tris, 0.15 M NaCl and 0.05% Tween 20 (pH 8.0) followed by incubation with pure monoclonal IgM antibody in different concentrations (100, 50, 25 10 and 1 µg/ml), 100 µl/well, in a buffer containing 0.1 M Tris, 0.3 M NaCl and 0.05% Tween 20 (pH 8.0) and incubated overnight at 4°C as previously described [22]. A positive control was used on each plate. The next day, plates were washed and incubated with alkaline phosphatase conjugated goat F(ab′)_2_ anti-mouse IgM (µchain)-specific conjugate (Jackson Immunoresearch Laboratories, Inc., The Netherlands), diluted 1∶2000 in incubation buffer for 1 hour at 37°C. After washing, 100 µl of freshly made substrate containing 1 mg/ml of nitrophenyl phosphate in di-ethanolamine buffer at pH 9.8 was added to each well. Plates were read at 405 nm.

#### Determination VH sequence of the monoclonal IgM antibody

To determine the VH sequence of the IgM monoclonal antibody, the heavy chain was cloned and sequenced after amplification with universal VH gene specific 5′ AGGTSMARCTGCAGSAGTCWGG 3′ [27] and a primer for the constant region of IgM 5′ [28]. The sequence was compared with germline VH genes by using the international ImMunoGeneTics information system/−QUEry and Standardization (IMGT/V-Quest) software program [29]. Following comparison with the IMGT reference directory, IMGT/V-QUEST identifies the variable (V), diversity (D) and Joining (J) germline alleles involved, determines the N-junctional diversity, and calculates the percentage nucleotide mismatches, suggestive for somatic hypermutation.

#### Collar-induced atherosclerosis

**Figure 1:**
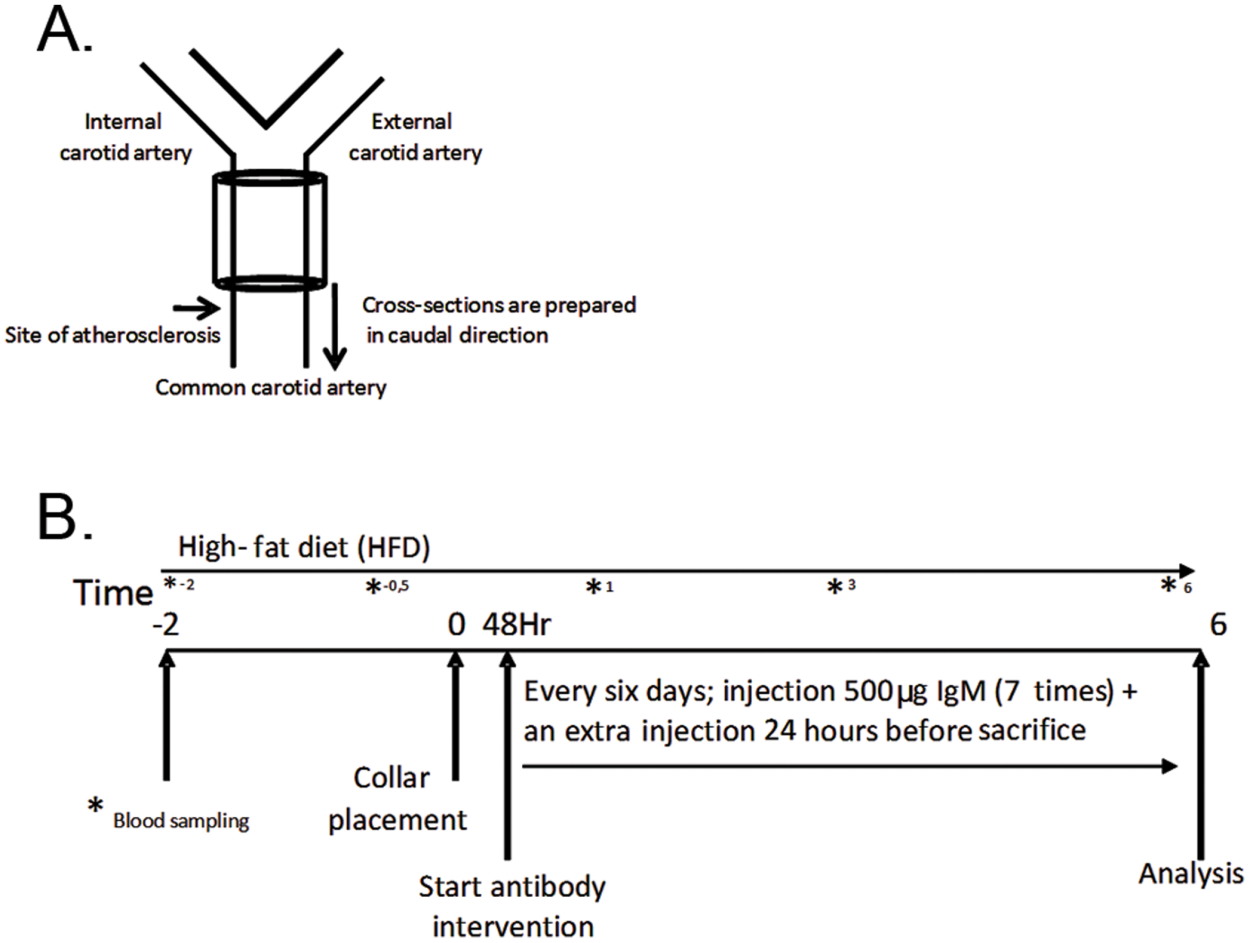
Schematic representation of the collar model and the experimental design. A. Atherosclerotic lesions develop caudal to the collar, cross-sections were made of the common right carotid artery in a caudal direction from the collar and collected in a parallel series of slides, B. Time schedule of the experiment to study the effect of passive immunization using Moab A7S8 or PBS on atherosclerotic plaque development.

Twelve-week-old male LDLr^−/−^ mice (n = 18 per group), obtained from the Jackson Laboratory, were operated after 2 weeks of high fat diet (0.25% cholesterol, 16% fat) to introduce a 2 mm long non-constrictive silastic tube around both carotid arteries, as described before [30]. Mice were anesthetized by 3 - 4% isoflurane and maintained by 1.5–2.5%. Pain relief was accomplished by a subcutaneous injection of buprenorphine 0.1 mg/kg preoperative and at the end of the day. During the 6 post-operative weeks, the high-fat diet was continued and the mice were treated every six days by an intraperitoneal injection with 500 µg Moab A7S8, starting from day two after collar placement. In addition, two control groups of mice were treated every six days with 500 µg PBS or a non-relevant IgM monoclonal antibody directed to human anti-EGP2/anti-epCAM (MOC153, a generous gift from prof. dr. L.F.M.H. (Lou) de Leij, University Medical Center Groningen, Groningen, The Netherlands). A final intraperitoneal injection was given 24 hour before sacrifice. In total 8 injections were given (see figure 1A,B for experimental design). The mice were sacrificed by carbon dioxide (gruadual fill) and were exsanguinated. Upon sacrifice the right carotid artery was isolated and embedded in paraffin. Sections of 5 mm were made and from every 100 mm, sections were stained with haematoxilin/eosin for lesion area analysis. After sacrifice, the heart and aorta were taken out, embedded in OCT (Sakura Finetek, Zoeterwoude, The Netherlands), and frozen on dry ice. The hearts were cut perpendicular to the heart axis just below the atrial tips. Sections of 7 µm were cut out of the heart in the area where the atrioventricular valves were visible. For lesion area measurements, four toluidin-stained sections, with an interval of 42 µm, were analyzed using Adobe Photoshop software, as described previously [31], [32].

#### Antibody measurement

To determine anti-oxLDL antibody titers, ELISAs were performed as described below. Blood samples were taken: before introduction HFD (T = −2), one day before collar placement (T = 0), one week after collar placement (T = 1; after 1× intervention with PBS or Moab A7S8), 3 weeks after collar placement (T = 3; after 5× intervention with PBS or Moab A7S8) and on day of sacrifice (T = 6; after 8× intervention with PBS or Moab A7S8).

#### Total IgM antibody

Total IgM and IgG levels were measured in a chemiluminescent-based sandwich ELISA using polyclonal goat anti-mouse IgM for capture and alkaline phosphatase (AP)-labeled goat anti-mouse IgM (µ-chain specific) for detection. Sera were diluted 1∶10.000 for total IgM.

#### MDA-LDL and copper-oxLDL antibody titers

Human LDL was isolated and MDA-LDL and copper-oxidized LDL were prepared as described [10]. Specific antibody titers were determined by chemiluminescent enzyme immunoassays as described [9], [33]. Briefly, sera were diluted 1∶100 and antibody binding to the indicated antigens was measured using the following secondary antibodies: alkaline phosphatase (AP)-labeled goat anti-mouse IgM (µ-chain specific) and IgG (γ-chain specific) (Sigma).

#### IgM - apoB immune complexes

ApoB-100 particles were captured on microtiter wells using LF3, a monoclonal antibody specific for murine apoB-100 which was coated on microtiter wells at 5 µg/ml in PBS. After washing and blocking steps, plasmas (1∶100 in BSA-PBS) were incubated in wells for 1 hour at room temperature and after further washing, bound IgM or IgG was detected using AP-conjugated goat anti-mouse IgM or anti-IgG by chemiluminescent ELISA. For detection of T15/EO6 antibodies bound to the captured apoB-containing particles, biotinylated AB1–2 was used, followed by incubation with AP-labeled NeutrAvidin and LumiPhos 530. In parallel wells, the relative amount of apoB captured in each sample was determined using biotinylated LF5, another monoclonal antibody specific for mouse apoB-100, followed by incubation with AP-labeled NeutrAvidin and LumiPhos 530. Because LF5 binds to only one epitope of apoB-100, the amount of each antibody used in this assay bound to the captured LDL was then normalized for the amount of captured apoB, and expressed as a ratio of antibody counts (RLU/100 ms) to apoB-100 counts (RLU/100 ms) [9].

#### Hypochlorite-oxLDL antibody titers

LDL was isolated from a plasma pool of healthy human subjects. Shortly, HOCl modification was performed by coating 100 µl of native LDL, diluted to 100 µg/ml in PBS, in microtitre plates (Nunc MaxiSorp™, Nalgene Nunc, Rochester, NY, USA) overnight at 4°C. After washing three times with PBS, half of each plate was incubated for 2 hours at 4°C in 36 µM sodium HOCl (Sigma, St Louis, MO, USA) in PBS; the other half of the plate was incubated in PBS. Incubation was followed by a washing with PBS to stop the reaction, and plates were stored in PBS at 4°C for a maximum of 2 h before starting the enzyme-linked immunosorbent assay (ELISA). Wells were then washed five times with a buffer containing 0.01 M Tris, 0.15 M NaCl and 0·05% Tween 20 (pH 8.0) followed by incubation in duplo with mouse plasma in a 1∶50 dilution, 100 µl/well, in a buffer containing 0.1 M Tris, 0.3 M NaCl and 0.05% Tween 20 (pH 8.0) and incubated overnight at 4°C. A positive control was used on each plate to test for intra-assay variation. The next day, plates were washed and incubated with alkaline phosphatase conjugated goat F(ab′)_2_ anti-mouse IgM (µchain)-specific conjugate (Jackson Immunoresearch Laboratories, Inc., The Netherlands), diluted 1∶2000 in incubation buffer for 1 h at 37°C. After washing, 100 µl of freshly made substrate containing 1 mg/ml of nitrophenyl phosphate in di-ethanolamine buffer at pH 9.8 was added to each well. Plates were read at 405 nm. Results are expressed as mean absorbance values (405 nm) from duplicate determinations and were calculated by subtracting binding to native LDL from binding to oxLDL.

#### EO6/T15 clonotypic antibody titers

For the detection of the natural IgM T15 clonotypic antibodies (e.g., EO6) in the plasma of mice, a chemiluminescent-based double antibody capture assay was used [10]. In this assay, a monoclonal anti-T15–idiotypic antibody AB1–2, a mouse IgG1, specific for both the canonical T15 VH and the T15 V_L_ regions was used as capturing antibody. For the assay, 2 µg/ml of AB1–2 in PBS was coated on microtitre plates overnight at 4°C. After washing and blocking steps, 50 µl of murine plasma diluted by 1∶100 in BSA-PBS was incubated overnight at 4°C. After further washing, captured T15-clonotypic antibodies were detected using an AP-conjugated goat-anti-mouse IgM (Sigma, St Louis, MO, USA) diluted in BSA-TBS, and a 50% aqueous solution of LumiPhos 530.

#### Collagen content

Picro-Sirius red staining was analyzed to determine collagen content within lesions in LDLr^−/−^ mice. Collagen positive area was determined from the largest atherosclerotic plaque of each mouse and analysed by expressing the stained surface area as a percentage of the total area of the plaque.

#### Plasma cholesterol

lasma samples for cholesterol determination were taken before start of the HFD, before collar placement and when mice were sacrificed. All plasma samples were taken after overnight fasting. Plasma cholesterol levels were measured with a commercially available kit (Sigma) following manufacturer’s instructions.

#### Statistical analysis

All statistical analyses were performed using GraphPad Prism (GraphPad Software Inc., San Diego, California, USA). Errors are shown as SEM and P<0.05 was considered as significant. Plaque volume measurements at the collar were done by a 2-way ANOVA. Differences between groups were analysed using a 1-way ANOVA when several groups were analysed; a two-tailed Student’s t-test or the Mann-Whitney U-test was used when comparing two groups with parametric or non-parametric data, respectively.

## Results

### Characteristics Monoclonal Antibody A7S8 (Moab A7S8)

**Figure 2:**
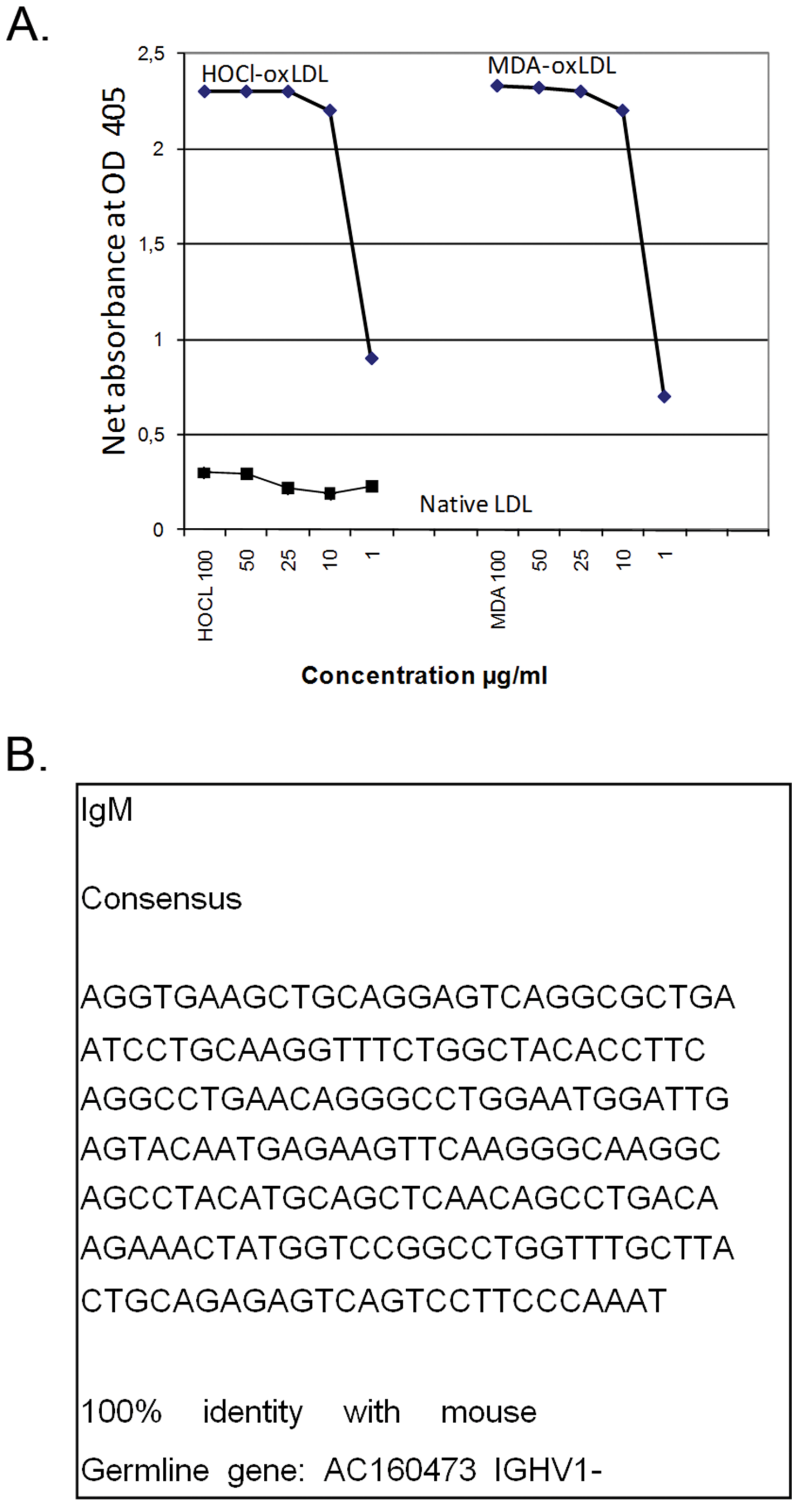
IgM Monoclonal antibody (Moab A7S8). A. Reactivity to native, HOCL-oxLDL and MDA-oxLDL. Measured by ELISA, OD 405 at different concentrations (100, 50, 25, 10 and 1 µg/ml IgM concentration). B. Sequence IgM hybridoma. The sequence of Moab A7S8 was compared with germline VH genes by using the international ImMunoGeneTics information system/−QUEry and Standardization (IMGT/V-Quest). Moab A7S8 is composed of IgHV1–78*01, IGHJ3*01 and IGHD1-1*02 alleles, with 100% identity and no gaps. Except for the N-addition, the alleles are in frame germline sequences.

To create an IgM monoclonal antibody to oxLDL, we immunized mice with human oxLDL. With ELISA techniques, we have selected and up scaled a monoclonal IgM antibody with specificity for HOCl-oxLDL, designated Moab A7S8. This Moab A7S8 was detected to be a pentameric IgM (κ) with no further degradation, which was shown by western blotting techniques by which a protein at approximately 800 kb (data not shown) was detected. The specificity of Moab A7S8 was shown using ELISA in which Moab A7S8 reacted strongly with HOCl-oxLDL and malonaldehyde (MDA)-oxLDL (Figure 2A). No reactivity in this assay was observed with Cu-oxLDL, phosphorylcholine or native LDL showing Moabs A7S8 specificity. The non-relevant control IgM showed no reactivity with HOCl-oxLD, MDA-oxLDL, Cu-oxLDL or native LDL (data not shown).

Moab A7S8 is composed of IgHV1–78*01, IGHJ3*01 and IGHD1–1*02 alleles, with 100% identity and no gaps. Except for N-addition, the alleles are in frame germline sequences, therefore B cell clone A7S8 produces a prototypical natural IgM antibody with an affinity for oxLDL (Figure 2B).

### Treatment with the A7S8 Antibody Result in a Decrease in Atherosclerotic Plaque Volume

**Figure 3:**
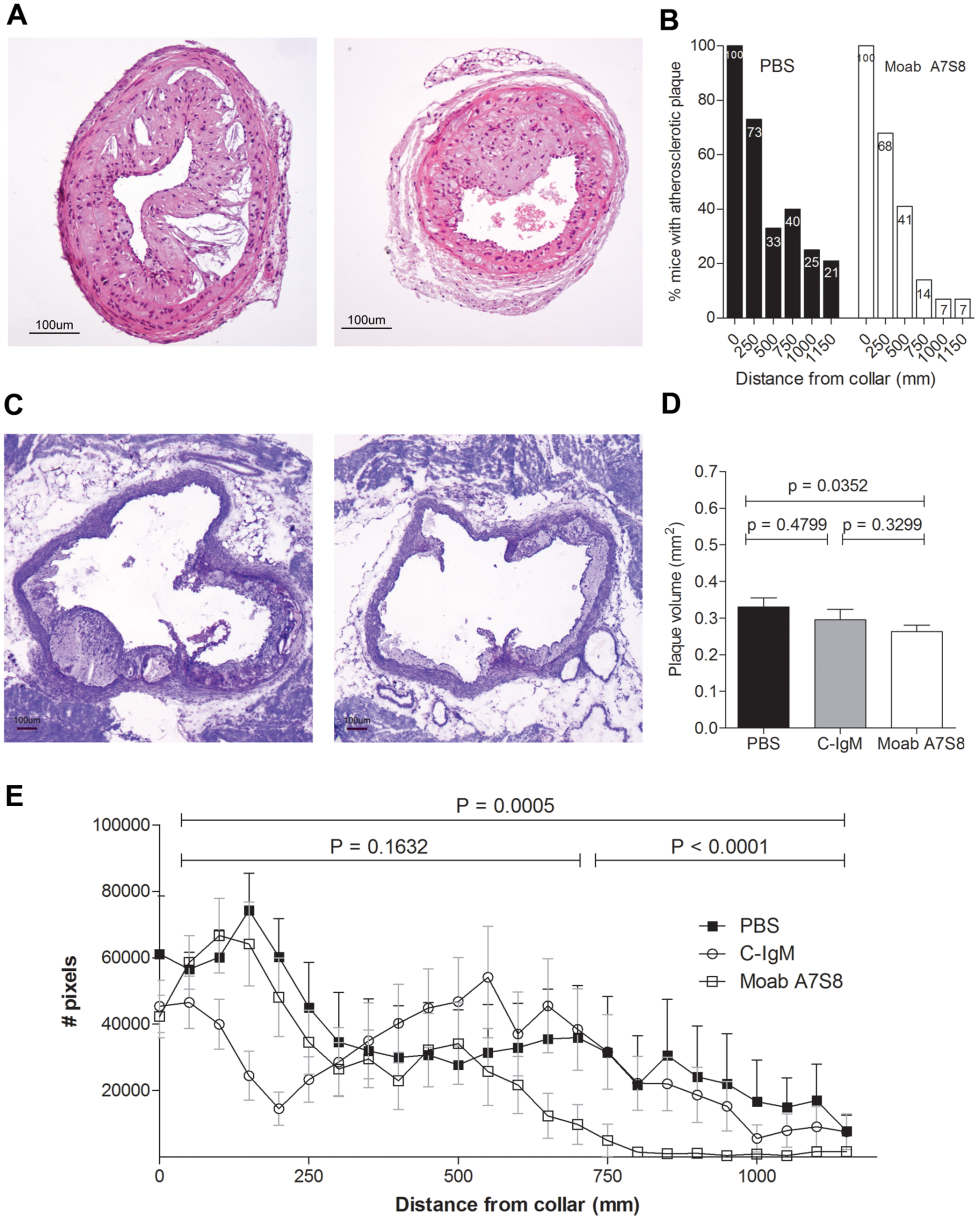
Atherosclerotic plaque volume at the collar and aortic root. A. Representative lesions of PBS and Moab A7S8 treated mice of collar-induce atherosclerosis in LDLr^−/−^ mice. Scale bar, 100µm (haematoxylin-eosin). B. Percentage mice with atherosclerotic plaque formation after certain distances from collar. Closed bars: PBS treated mice. Grey bars: control IgM treated mice. Open bars: Moab A7S8 treated mice. C. Representative lesions in the aortic root of PBS and Moab A7S8 treated LDLr^−/−^ mice. Scale bar, 100 µm (toluidin blue). D. Atherosclerotic plaque volume in the aortic root. Plaque volume, expressed in mm2. E. Collar-induced atherosclerotic plaque volume, measured at every 50 µm from collar, in PBS (▪), control IgM (○) and Moab A7S8 (□) treated mice. A dot represents the mean ± SEM.

To evaluate the effect of a passive immunization strategy using our Moab A7S8 with specificity for HOCl-oxLDL on the development of atherosclerosis, LDLr^−/−^ mice were 8 times injected intraperitoneally with Moab A7S8, control-IgM or PBS. At sacrifice, plaques were located in the proximal area to the collar and in the aortic root. Atherosclerotic plaque formation in the right carotid artery was analyzed using haematoxilin-eosin staining. Treatment of anti-oxLDL natural IgM antibodies resulted in a significant reduced plaque volume formation at the collar (p = 0.002, figure 3A,B,E) in atherosclerotic mice. Mice injected with PBS or control-IgM develop a plaque volume which reaches to the 1150 µm distance from the collar. In contrast, mice treated with anti-oxLDL IgM antibodies develop plaque content to a distance of 750 µm from the collar (Figure 3B,E). At 750 µm from the collar, only 14% of the Moab A7S8 treated mice show any plaque development in the carotid artery, whereas 40% of the PBS treated mice demonstrated plaque formation at that site (Figure 3D,E). Beyond 750 µm distance from the collar, plaque formation was virtually blocked by the treatment with Moab A7S8 as shown by the highly significant effects at these distances. At the aortic root, the average plaque size measured in the mice treated with PBS, control-IgM and Moab A7S8 was respectively 330 µM^2^ (SEM 25), 295 µM^2^ (SEM 28) and 263 µM^2^ (SEM 17) (figure 3C,D). The reduction in plaque size after treatment with Moab A7S8 was significant when compared to PBS treatment (p = 0.0352), while non-significant compared to control-IgM treatment (p = 0.3299).

**Figure 4:**
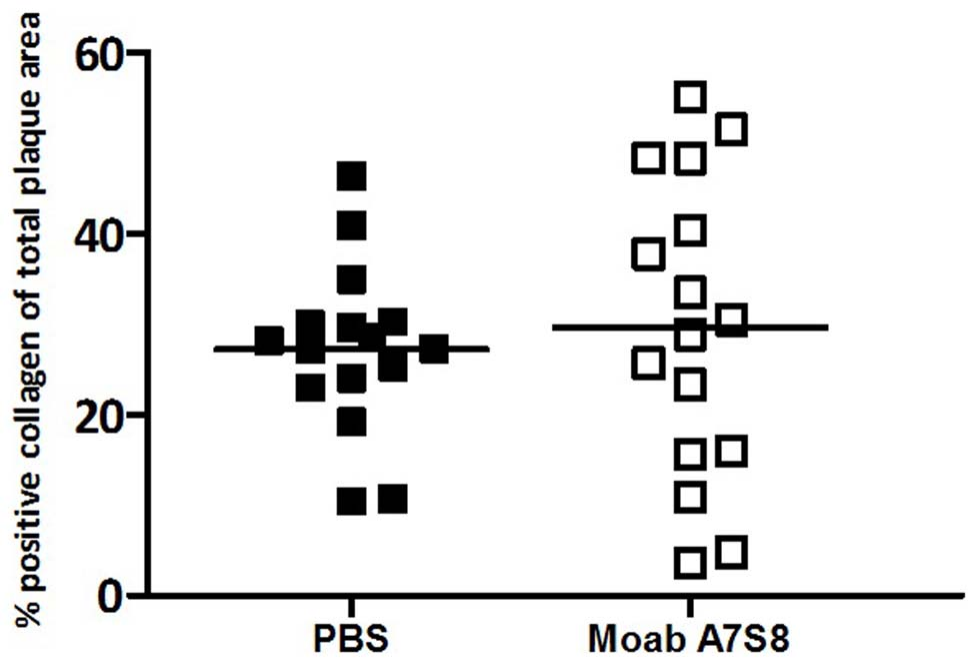
Collagen content in the atherosclerotic plaque. Percentage positive collagen of the total plaque area. In PBS (▪) and Moab A7S8 (□) treated mice.

**Figure 5:**
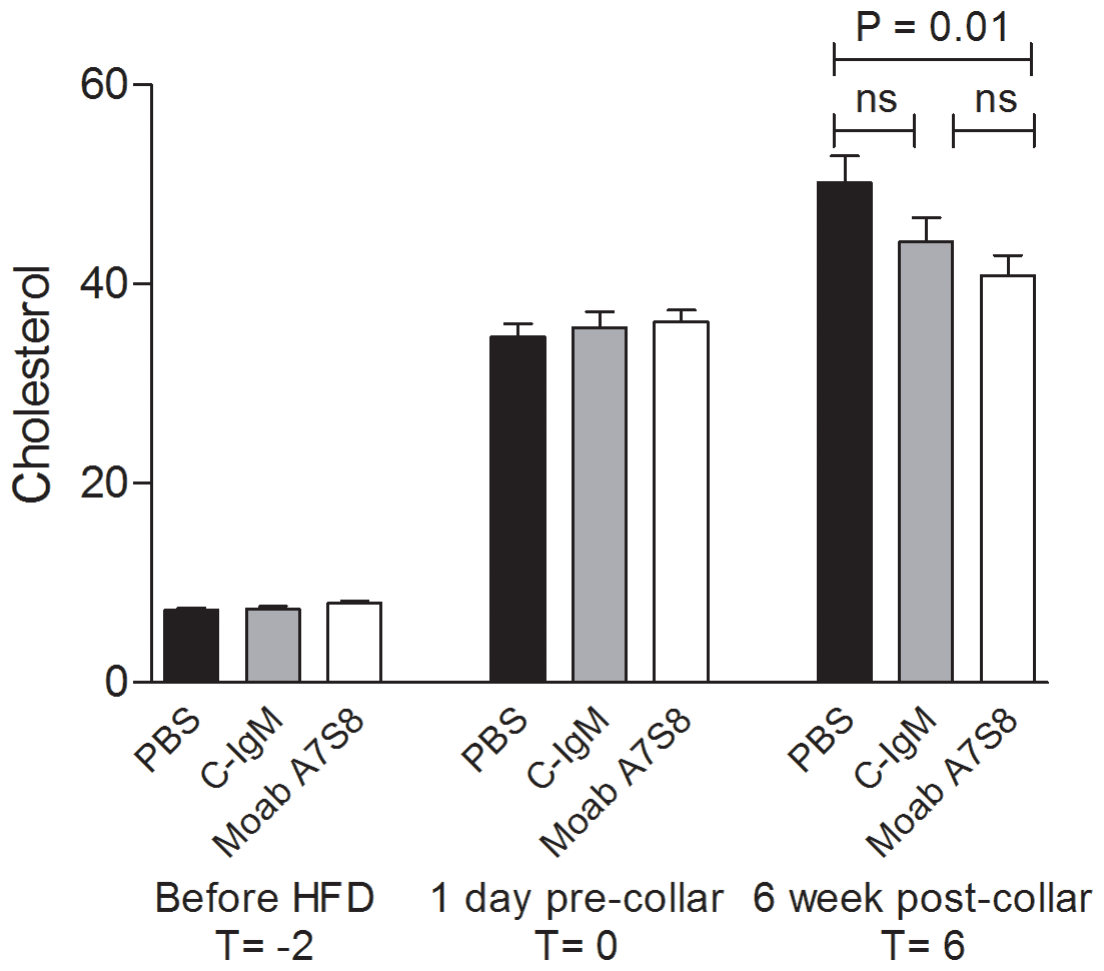
Cholesterol levels. Serum cholesterol levels are expressed as mM (± SEM). Closed bars: PBS treated mice. Grey bars: control IgM treated mice. Open bars: Moab A7S8 treated mice.

As shown in Figure 4, there was no difference between mice treated with PBS or treated with Moab A7S8 in total collagen content of their plaques (PBS: 27% ±13 and Moab A7S8∶29% ±18; P = 0.588). Total cholesterol levels and body weight were measured at three different time points during the experiment. During the experiment, PBS, C-IgM and Moab A7S8 treated mice did not differ in body weight (weight in grams at sacrifice: PBS: 23±0.4, C-IgM: 23±0.2, Moab A7S8∶23±0.2). Cholesterol levels were the same at baseline (T = −2) and before carotid collar placement (T = 0). At sacrifice, however, mice treated with Moab A7S8 showed a significant (P = 0.01) 20% lower plasma cholesterol compared to mice injected with PBS (PBS: 49±8 mM/l; Moab A7S8∶41±8.5 mM/l, see figure 5). Compared to C-IgM treatment, plasma cholesterol levels in the Moab A7S8-treated group were also lower, although not statistically significant (C-IgM: 44±10.5 mM/l).

### Total IgM and anti-oxLDL IgM Antibody Levels

**Figure 6:**
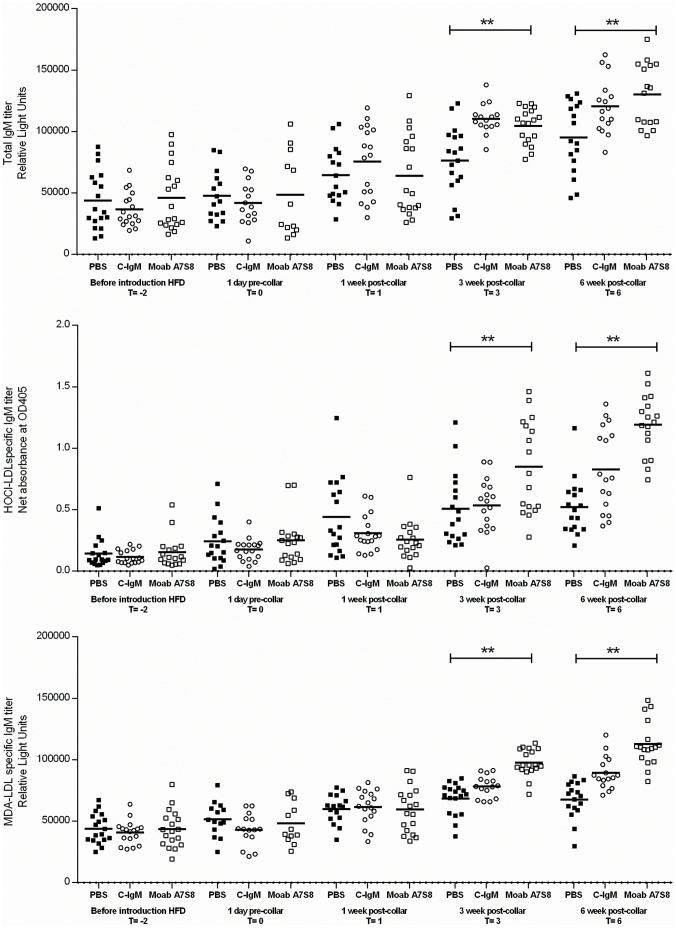
Total IgM and HOCl and MDA anti-oxLDL IgM titers. A. Total IgM titers, B. anti-HOCl oxLDL IgM titers and C. anti-MDA oxLDL IgM in PBS treated mice (▪), control IgM treated mice (o) and Moab A7S8 treated mice (□).

At T = −2 and T = 1 (in weeks), no differences could be observed in total IgM titers, HOCl-oxLDL and MDA-oxLDL IgM levels in mice treated with PBS or Moab A7S8. However, after 5 injections (T = 3) of Moab A7S8 antibodies a significant increase in total IgM, HOCl-oxLDL and MDA-oxLDL IgM antibodies could be observed compared to the PBS treated group. The total IgM levels also increased after this point in time in the PBS and C-IgM group, but to a lower extent compared to the Moab A7S8 treated group. Further, it could be observed that the difference between the A7S8 and the control groups were higher for the HOCl-oxLDL and MDA-oxLDL IgM antibodies compared to total IgM. Comparable but more pronounced differences could be observed after 3 more injections (T = 6) (Figure 6).

**Figure 7:**
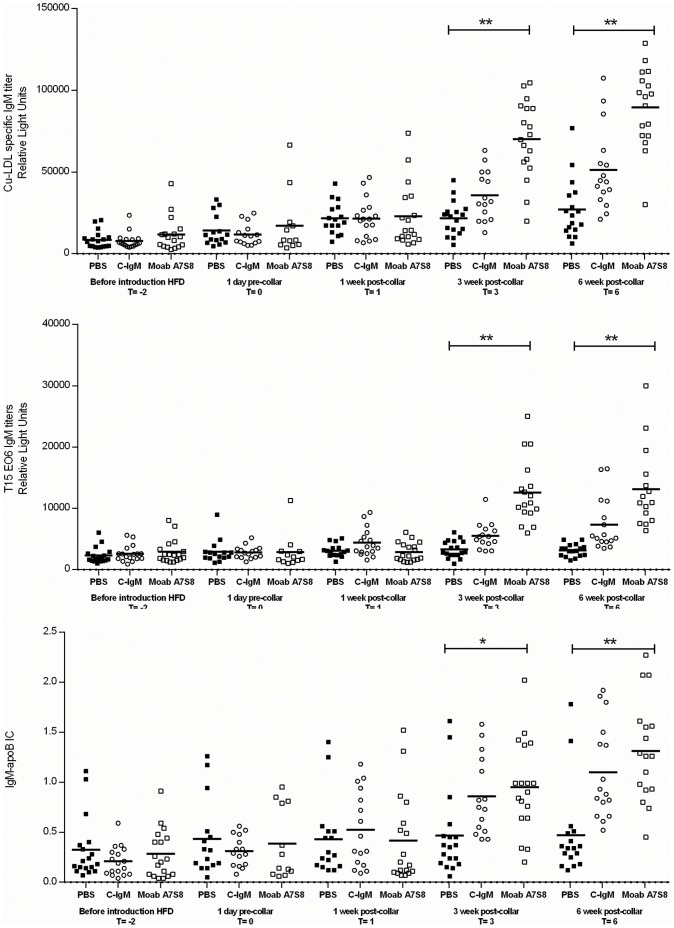
Cu-anti-oxLDL IgM titers, T15/EO6 IgM antibodies and IgM-apoB immune complexes. A. Anti-Cu-oxLDL IgM titers, B. T15/EO6 IgM antibodies and C. IgM-apoB immune complexes expressed as IgM/apoB. Mice treated with PBS (▪), control IgM treated mice (o) and in mice treated with Moab A7S8 (□).

Also, after 3 weeks and 6 weeks of Moab A7S8 treatment, Cu-oxLDL IgM, T15 IgM antibodies and IgM-apoB Immune complexes increased dramatically (Figure 7). These very specific effects that occur beyond 3 weeks of treatments clearly show the active biological consequences of the passive immunization treatment with Moab A7S8.

### Anti-oxLDL IgG Levels

**Figure 8:**
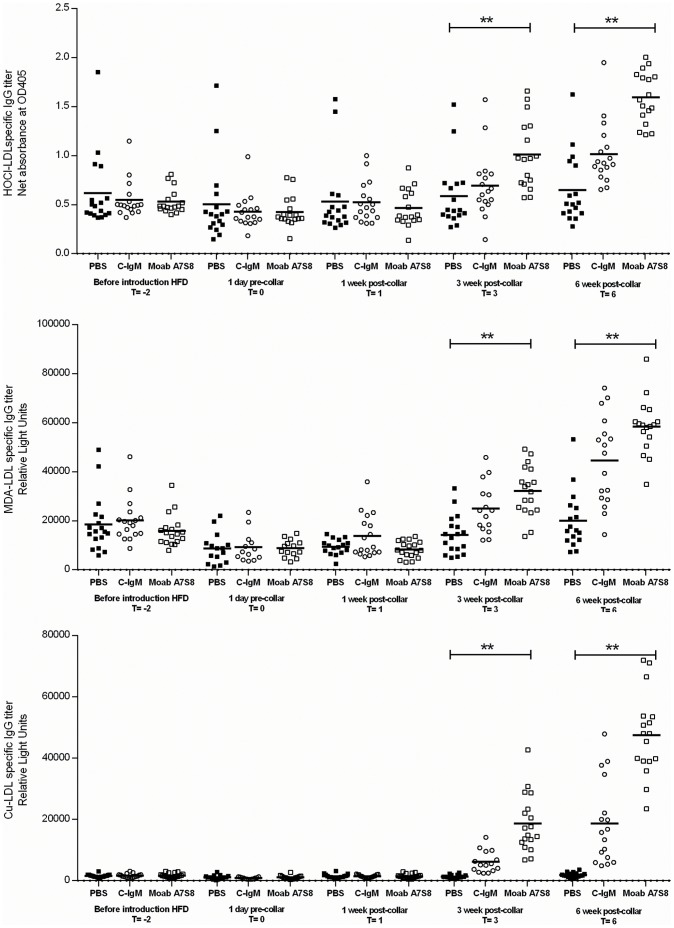
Anti-HOCl, MDA and Cu-oxLDL IgG titers. A. Anti-HOCl-oxLDL IgG titers, B. anti-MDA-oxLDL IgG titers and C. anti-Cu-oxLDL IgG titers in mice treated with PBS (▪), control IgM treated mice (o) and in mice treated with Moab A7S8 (□).

At T = −2, T = 0 and T = 1 (in weeks), no differences could be observed in HOCl-oxLDL IgG, MDA-oxLDL IgG and Cu-oxLDL IgG titers. However as also was demonstrated for the IgM anti-oxLDL antibodies, in addition, also IgG titers against HOCl-oxLDL and MDA-oxLDL significantly rose after 3 and 6 weeks of Moab A7S8 treatment (Figure 8).

## Discussion

This study demonstrates that passive immunization with Moab A7S8 directed against HOCl-oxLDL results in decreased atherosclerosis development. By injecting Moab A7S8, we observed in our collar model a significant decrease of plaque development compared to PBS treatment. During lesion development, cholesterol levels decreased and anti-oxLDL IgM and IgG titers increased.

Several modifications of LDL into oxidized forms of LDL (oxLDL) can occur during atherosclerosis development. Hypochlorite (HOCl) is the product of the H_2_O_2_-catalyzed oxidation of chloride by the enzyme myeloperoxidase (MPO). HOCl is highly reactive and can result in the oxidation of the LDL particle [16]. Modification of LDL by HOCl is an important *in vivo* oxidation pathway since MPO, released from plaque-infiltrating neutrophils and monocytes, is abundantly present in atherosclerotic plaques [17], [24]. Importantly, several studies have furthermore implicated a role for MPO in human cardiovascular diseases [18]–[20], [34].

By VH sequencing, we could determine that our Moab A7S8 is a natural antibody. With their ability to recognize self, altered self and foreign antigens, natural antibodies comprise an important first-line defense against invading pathogens, but are also important for tissue homeostasis [6], [7]. Recently, Chou et al. [33] demonstrated that oxidation-specific epitopes constitute a dominant target for natural antibodies. Approximately 30% of all IgM-secreting clones produce natural antibodies that can bind to oxidation-specific epitopes [33]. Inflammatory events, as in atherosclerosis occurs, are associated with enhanced oxidative stress, and different oxidation-specific antibodies are induced not only during atherogenesis, but also in a variety of other inflammatory settings [35], [36]. Therefore, it was no surprise that our Moab A7S8 IgM appeared to be part of these natural antibodies recognizing oxidation-specific epitopes. Previously, it has been shown that another natural antibody EO6/T15 is athero-protective, in part through inhibiting the uptake of oxLDL by macrophages [10]. Chou et al. [33] have shown that another natural antibody, clone NA-17, has also the ability to inhibit the uptake of MDA-oxLDL by macrophages in a similar manner as the T15/EO6 antibody. This mechanism has also been demonstrated in humans where anti-phosphorylcholine IgM could inhibit the ex vivo uptake of modified LDL by macrophages [37]. Therefore, it is not unlikely that our Moab A7S8 confers also these abilities to inhibit uptake of oxLDL by macrophages. Moab A7S8 differs from the EO6/T15 antibody in VH gene usage: Moab A7S8 uses the IgHV1–78*01 VHgene that is a member of the J558 (V1) VH gene family, whereas EO6/T15 uses the S107.1 VH gene. Therefore, we postulate that our Moab A7S8 recognizes other epitope(s) than the EO6/T15 antibody, and possibly could have other (stronger) effects on atherogenesis. Epitope mapping of our Moab A7S8 against HOCl-oxLDL might be important to further define antigenic epitope(s).

An athero-protective role of anti-oxLDL antibodies was demonstrated in studies in which treatment with different PC-specific related molecules reduced atherosclerotic plaque development. First, passive immunization with PC-specific T15 IgM antibodies reduced vein graft atherosclerosis in ApoE^−/−^ mice [5]. Secondly, immunization with streptococcus pneumonia vaccine, which contain the PC-epitope is associated with an increase in PC-specific antibodies and reduced atherosclerosis [10]. Also, immunization of ApoE^−/−^ mice with PC linked to a carrier protein resulted in inhibition of atherosclerosis [38]. This latter approach resulted in the generation of both PC-specific IgM as IgG antibodies and it was suggested that these antibodies facilitates the removal of oxLDL from the arterial extracellular tissue (“reverse cholesterol transport”) [38].

Interestingly, our control IgM reduced the plaque volume at the aortic root, although this difference did not reach significance. These findings are in accordance with Cesena et al. [39], who showed that polyclonal IgM reduces atherosclerosis in apoE−/− mice. Immunoglobulin therapy is known to have multiple effects on immune function, including enhancing the auto-antibody repertoire and down-modulating T cell function. Indeed, we could confirm raised auto-antibodies in the control IgM group compared to the PBS group. Even though our control monoclonal antibody did not show reactivity against oxLDL as tested by ELISA, the increase of antibodies to oxLDL and decrease of serum cholesterol levels after treatment with our control monoclonal antibody makes the results obtained by our control antibody difficult to interpret.

In our study, we observed a 20% decrease in cholesterol levels in Moab A7S8 treated mice. Decreased cholesterol levels contributed probably to the decreased atherosclerotic plaque development caused by passive immunization with Moab A7S8. We postulate that large amounts of IgM antibodies formed immune complexes with (minimally) oxLDL in plasma and resulted in enhanced clearance of oxLDL. During Moab A7S8 treatment IgM-ApoB immune complexes were strongly induced in our mice. In the pneumococcal vaccination study lower cholesterol levels were also found in the vaccinated mice in comparison with the PBS treated mice [10]. In the study by Fario-Neto, however, while using T15 monoclonal antibodies no decreased levels of circulating cholesterol were found [5]. Not only IgM anti-oxLDL levels increased during the Moab A7S8 therapy as result of passive immunization but interestingly also specific anti-oxLDL IgG levels increased indicating an immune modulatory phenomenon. When IgM is injected it may bind to oxLDL in the circulation, resulting in immune complex formation. These immune complexes could, subsequently, be taken up by antigen presenting cells (APCs). These APCs present the oxLDL antigen to antigen specific Thelper cells which could subsequently result in specific B cell activation resulting in an increased anti-oxLDL IgG production. Presentation of PC-specific motifs by APCs has been shown to to be important in the generation of athroprotective effects. Vaccination with ox-LDL reduces plaque development during atherogenesis [40] and, importantly, ox-LDL pulsed dendritic cell vaccinations resulted in 87% reduction in carotid artery lesion size in the same atherosclerosis model that was used in the current study. In a study by Ehrenstein et al. [41], it was shown that natural IgM antibodies could play a role in facilitating an acceleration of the primary immune response. Immune complexes consisting of natural IgM antibodies may activate complement and consequently may cause effective B cell activation. Indeed, it has been demonstrated that the primary immune response can be accelerated by the administration of specific IgM antibody. Unfortunately, in the study of Faria-Neto et al. [5] anti-oxLDL antibody levels in mice treated with natural T15 IgM antibodies were not measured. Whether administration of (natural) IgM antibodies and subsequently induction of IgM and IgG oxLDL specific titers directly causes reduced carotid plaque development in humans remains to be elucidated.

In conclusion, this study demonstrates an inhibition of atherosclerotic plaque formation by an intervention with Moab A7S8 specific for HOCl-oxLDL. Treatment resulted in decreased cholesterol levels and induction of specific IgM and IgG titers against oxLDL. This evidently shows the active biological consequences of the passive immunization strategy using Moab A7S8 in mice. Further research should be focussed on identification of the antigenic epitope(s) on HOCl-oxLDL which could induce these specific IgM and IgG antibody response against oxLDL. We postulate that therapeutic effects of IgM treatment should be further studied as a potential intervention for human cardiovascular diseases.
